# Gene by Culture Effects on Emotional Processing of Social Cues among East Asians and European Americans

**DOI:** 10.3390/bs8070062

**Published:** 2018-07-11

**Authors:** Arash Javanbakht, Steve Tompson, Shinobu Kitayama, Anthony King, Carolyn Yoon, Israel Liberzon

**Affiliations:** 1 Department of Psychiatry and Behavioral Neurosciences, Wayne State University, 3901 Chrysler Service Drive, 3rd Floor, Detroit, MI 48201, USA; 2 Department of Psychiatry, University of Michigan, 4250 Plymouth Rd., Ann Arbor, MI 48109, USA; samadhi@med.umich.edu (A.K.); liberzon@med.umich.edu (I.L.); 3 Department of Psychology, University of Michigan, 3217 East Hall, Ann Arbor, MI 48109, USA; tompson@umich.edu (S.T.); kitayama@umich.edu (S.K.); 4 Stephen M. Ross School of Business, University of Michigan, 701 Tappan Ave, Ann Arbor, MI 48109, USA; yoonc@umich.edu

**Keywords:** culture, gene, emotion, social, amygdala, insula

## Abstract

While Western cultures are more focused on individualization and self-expression, East Asian cultures promote interrelatedness. Largely unknown is how gene by culture interactions influence the degree to which individuals acquire culture, and the neurocircuitry underlying how social cues are processed. We sought to examine the interaction between *DRD4* polymorphism and culture in the neural processing of social emotional cues. 19 Asian-born East Asian (AA) and 20 European American (EA) participants performed a shifted attention emotion appraisal functional magnetic resonance imaging (fMRI) task, which probes implicit emotional processing and regulation in response to social emotional cues. Half of the participants in each group were *DRD4* 2- or 7-repeat allele (2R/7R) carriers. AA participants showed larger left and right amygdala, and left hippocampal activation during implicit processing of fearful faces. There was a gene by culture interaction in the left insula during implicit processing of facial cues, while activation in EA *DRD4* 2R/7R carriers was larger than EA non-carriers and AA carriers. Our findings suggest that emotional facial cues are more salient to AA participants and elicit a larger amygdala reaction. Gene by culture interaction finding in insula suggests that *DRD4* 2R/7R carriers in each culture are more prone to adopting their cultural norm.

## 1. Introduction

There is accumulating evidence about the brain areas involved in implicit and explicit emotion regulatory functions that are highly needed for social interactions [1,2]. Among the implicit functions, there is attention-related regulation when shifting attention to a neutral stimulus or a cognitive task that reduces amygdala response. Implicit emotion regulation also happens with labeling or the appraisal of one’s emotional response to an emotion-provoking stimulus. Appraisal of one’s emotions, which engages the prefrontal cortical areas, reduces emotional reactivity in the amygdala. Explicit emotion regulation happens when a participant is asked to dampen or change the emotional response. A strategy that participants commonly use in such cases is re-appraisal, through which the participants cognitively re-appraise the stimulus, or create a different narrative for it.

A decisive factor in emotional processing style and extent is culture and its differences. For example, people who are from Western cultures tend to endorse and internalize beliefs and values that emphasize the individual as distinct from others and are defined by internal traits, and value expressing their unique attributes (called independent social orientation). On the other hand, people from East Asian cultures such as China or Japan tend to endorse and internalize beliefs and values that emphasize the individual as fundamentally connected to others and defined by relational traits, and value promoting and maintaining social harmony (called interdependent social orientation) [3]. This leads to individuals from Asian cultures tending to value emotional control [4] and thus they down-regulate their emotions using emotion suppression strategies [5]. In an interdependent culture, emotion has a more relational role and the person should be sensitive to, and take perspective of the other, to be able to maintain a connection to the group [3]. Furthermore, some have argued that because negative emotions are less frequently expressed in Asian cultures, their expression may signal a more negative situation [3,6]. In contrast, individuals from Western cultures tend to value emotional expression [4], view suppressing emotion as unhealthy [7], and prefer to re-appraise rather than suppress negative emotions [3,5]. In summary, while in Western cultures an awareness of one’s own emotions are rewarded, in East Asian cultures the person is more encouraged to be attuned to the emotions of others and social emotional cues [5].

A recent focus of neuroscience research has been on the neurobiological correlates of the impact of cultural differences on emotion processing. Levenson and colleagues showed that mimicking the same facial expression could elicit stronger self-reported emotions in Caucasian Americans than people from West Sumatra [8]. High-aroused emotions with stronger somatic feedback were also found to be more valued in Western than East Asian cultures [9]. Interestingly, activation in the somato-sensory cortex correlated with the strength of the emotions found in Caucasian Americans, but not in Chinese people. A recent meta-analysis also revealed higher insula activity in people from Western cultures compared to East Asians [10]. More emotionally expressive people also show a closer correlation between strength of experienced emotion and activation in the insular cortex [11]. Altogether, higher insular activation in Western cultures may be a correlate of higher affinity with one’s emotions.

Yet another level of complexity in the interplay between culture and neurobiology is the genetic differences among members of each culture, and its impact on differences in the neural processing of emotions. For instance, recent findings on the interplay between biological and environmental factors suggest that dopaminergic genes might shape brain activity to moderate cultural differences in psychological tendencies. The *DRD4* VNTR polymorphism involves a sequence of amino acids in the Exon 3 of the *DRD4* gene that repeats itself anywhere from 2 to 11 times, and the 2-repeat and 7-repeat versions of this gene are linked to higher levels of dopaminergic signaling capacity in the brain [12]. Carriers of the 2-repeat and 7-repeat versions of the *DRD4* VNTR polymorphism tend to exhibit heightened sensitivity to reward and external reinforcement [13,14], and therefore this gene may lead people to be more sensitive to reinforcement for culturally sanctioned behaviors. In a previous work we showed that Americans who were born and grew up in the United States and carry these high dopamine signaling alleles (2-repeat or 7-repeat alleles) were more independent, whereas Asians who were born and grew up in an East Asian country and carry the same alleles were more interdependent [15]. Interestingly, for individuals who do not carry the high dopamine signaling alleles (non-carriers), there was no cultural difference in independent (vs. interdependent) social orientation. This work suggests that individuals carrying *DRD4* VNTR polymorphism are more likely to endorse culturally normative beliefs and values. This finding had led us to propose a cultural norm sensitivity hypothesis [16]. This theory suggests that the degree to which individuals acquire cultural norms can be affected in both directions by the polymorphic variants of genes involved in dopaminergic neural pathways.

Collectively, previous work suggests that people of Western cultures might be reinforced to experience, express or reappraise emotional responses, while Asians might be reinforced to suppress emotional experiences and to be sensitive to the emotional experiences of others. Genetic make up might facilitate these alternative coping styles. What is yet unknown, however, is how the neurocircuitry of emotional processing mediates these effects.

In the current study, we sought to examine those neural mechanisms underlying gene × culture interaction. We use a validated functional magnetic resonance imaging (fMRI) paradigm to test whether genes and culture influence the neural processing of emotional faces. Based on the previous studies, we expected to see differences in emotion processing and its awareness (amygdala, hippocampus, insula), and emotion regulation (prefrontal cortex). We expected to see that Asians would show a larger emotional reactivity to the negative emotions of others. Furthermore, we hypothesized that European Americans would show a more insular activation (an index of awareness of the emotional response) in response to social emotional cues. Finally, we hypothesized that carriers of the 2-repeat or 7-repeat alleles *DRD4* VNTR polymorphism will show greater cultural differences in the neural processing of emotional faces than non-carriers, as they are more sensitive to cultural rewards.

## 2. Materials and Methods

### 2.1. Participants

A total of 39 people, 20 European Americans (EA) and 19 Asian-born Asians (AA), participated in this study. Participants were recruited through email and phone calls using a database of participants who had previously provided saliva samples for genetic testing. Demographics for this larger database are described previously [15]. In summary, we selected an equal number of carriers and non-carriers in each group. The larger cohort was nearly two third females (more so in carriers), and similarly there are more females in this study. A large number of participants from the original study were not available, as they had graduated by the time of recruitment for this work. The EA participants were all of European ancestry and were born and raised in the United States. To qualify as an AA, participants had to have been born and raised in an East Asian country, and not have spent more than 10 years in the United States. The gender distribution was 5F/5M EA non-carriers, 9F/1M EA carriers, 6F/4M AA non-carriers, and 7F/2M AA carriers. The average age was 23.35 ± 0.74 years for the EA group, and 23.79 ± 1.78 years for the AA group. Half of the participants in each group (9 EA, and 10 AA) were carriers of the 2-repeat or 7-repeat alleles *DRD4* VNTR polymorphism, and the other half (10 EA, and 10 AA) were non-carriers. This study was approved by the University of Michigan IRB with written informed consent from all subjects. All subjects gave written informed consent in accordance with the Declaration of Helsinki.

### 2.2. Genotyping

Oragene Saliva kit OG-500 was used for saliva collection (DNA Genotek, Ottawa, ON, Canada). Genomic DNA was extracted using a high-capacity membrane-based column (QuickGene810, AutoGen, Inc., Holliston, MA, USA), and was quantitated using A260/A280 ratio (Nanodrop), and agarose gel electrophoresis. The *DRD4* variable number tandem repeat (VNTR) polymorphism was amplified using 0.2 µM of each primer *DRD4* forward 5′-GCGACTACGTGGTCTACTCG and *DRD4* reverse 5′-AGGACCCTCATGGCCTTG [17], using the Roche “GC-Rich PCR System” amplification buffer (Roche Applied Science, Inc., Mannheim, Germany) and 20 ng of genomic DNA in a volume of 25 µL. The samples were heated in a Stratagene thermocycler (Life Technologies, Inc., Grand Island, NY, USA) at 95 °C for 3 min, then cycled 40 times at 95 °C for 20 s, 57 °C for 20 s, and 72 °C for 1 min, followed by 72 °C for 3 min. PCR products were separated and visualized on a 2% agarose gel (type 1-A, Sigma, St. Louis, MO, USA) stained with ethidium bromide.

### 2.3. Experimental Task Shifted-Attention Emotion Appraisal Task (SEAT)

In order to examine potential neural mechanisms underlying implicit emotional regulation, we used the SEAT paradigm. The SEAT was designed to examine implicit emotion processing and implicit emotion regulation by attention and by appraisal [18,19,20]. Previous studies have confirmed the validity of SEAT and of similar tasks in examining attention- and appraisal-related implicit emotion regulation [18,19,20,21]. The SEAT has been used to study how the brain processes emotional faces, and importantly, how environmental factors including childhood poverty and stress, as well as biological factors such as glucocorticoids and neurosteroids influence the neural processing of emotional faces. These studies demonstrated that the SEAT is well suited to test the neural circuits involved in implicit emotional regulation that might mediate interplay between biological and environmental factors. The detailed description of the task is available in previous publications, and here we describe it briefly.

Participants viewed pictures of fearful or neutral (45 of each) European American faces superimposed on background indoor or outdoor scenes. They were asked to determine one of the three following: (1) the gender of the face (Gender: implicit emotional response to the affect presented on the face). Here, participants implicitly process the emotional expressions of the faces, as it has shown that focusing on the gender facilitates the implicit processing of the emotion presented by the face [18,19,20]. In other words, the attention is focused on the emotional face when one is identifying the gender; (2) If the background scene is indoor or outdoor, (In/Out: implicit attention-related emotion regulation by shifting attention away from emotional faces and reducing emotional reactivity); or (3) appraise their own emotions and see if they like or dislike the emotion presented on the face thus triggering appraisal-related regulation of the emotional response (Like/Dislike: appraisal-related emotion regulation).

There were 3 runs and a total of 120 trials (40 trials for each run): 30 Gender, 30 In/Out, and 30 Like/Dislike. The remaining 30 trials were pictures of a neutral face or a place, and participants were asked to determine if it was a picture of a face or a place (Face/Place). Trials were presented in random order and consisted of presentations of a crosshair for 2000–5000 ms, a task cue for 750 ms, and a composite image for 1500 ms. The task is presented in the Supplementary Figure S1.

### 2.4. Behavioral Analyses

To examine the accuracy of the responses, we first computed the percentage of correct responses for the Gender, In/Out, and Face/Place trials for each participant. Since the Like/Dislike trial involved a subjective evaluation of faces, we did not compute accuracy for this task. Average accuracy rates were entered into a 2 (Culture: EA, AA) × 2 (Gene: carrier, non-carrier) ANOVA to assess for main effects and interaction effects. Follow-up simple effect analyses were performed with two-tailed *t*-tests, with a significance threshold set to 0.05.

To examine response times we first log-transformed the response time for each trial and then computed the average response time for the Gender, In/Out, Like/Dislike, and Face/Place trials for each participant. Average response times were entered into a 2 (Culture: EA, AA) × 2 (Gene: carrier, non-carrier) ANOVA to assess for main effects and interaction effects. Follow-up simple effects analyses were performed with two-tailed *t*-tests, with the significance threshold set to 0.05.

### 2.5. Acquisition of fMRI Data

All scanning was performed using a Philips 3 Tesla MRI scanner (Phillips Medical Systems, Andover, MA, USA) in the functional MRI laboratory at the VA Ann Arbor. A total of 240 T2*-weighted echo planar gradient-recall echo volumes (echo time = 30 ms, repetition time 2000 ms, 64 × 64 matrix, flip angle = 90 degree, field of view = 22 cm, 42 contiguous 3 mm axial slices per volume), were acquired. Five additional volumes were discarded at the beginning of each run to allow for equilibration of the MRI signal. A high-resolution T1-weighted structural image was also obtained to provide for more precise anatomical localization.

### 2.6. Functional MRI Data Analysis

Data were analyzed using the statistical parametric mapping software package, SPM8 (Welcome Department of Cognitive Neurology, London, UK). Functional volumes were slice time corrected to account for the temporal differences in slice acquisition time, realigned to correct for head motion, and spatially normalized to a standard template based upon the Montreal Neurological Institute (MNI) reference brain using a VBM8 toolbox and DARTEL high dimensional warping, and spatially smoothed using a 5-mm Gaussian kernel. Single-subject analysis was performed using fixed effects models within the general linear model as implemented in in SPM8.

Data were modeled using an event-related design and included separate boxcar functions for trials (Face, Place, Gender, In/Out, Like/Dislike) and task cues. All models also included nuisance regressors consisting of the motion correction parameters and their derivatives from the realignment preprocessing step. Following previously published methods [18,19,20], first-level contrasts included Gender > Face/Place (implicit emotional processing), In/Out > Gender (attention-related emotion regulation), and Like/Dislike > Gender (appraisal-related emotion regulation). Face/Place contrast was chosen as the baseline for the Gender contrast because it averages response to neutral faces and places, and hence what is measured in the contrast Gender > Face/Place is the emotional response to the fearful faces. Movement parameters were included as covariates for all models. All the subjects’ data were entered into a second-level general linear model controlling for age and gender. Areas of activation in each of the above contrasts at a whole brain FWE corrected *p*-value threshold of 0.05, and minimum number of 50 voxels in each cluster were identified (except for the occipital lobe that was not an area of interest). Spheres of 10 mm diameter were created around the voxel of maximum activation in each cluster, in each contrast, to extract betas. Because of the established role of the amygdala and hippocampus in salience detection and emotional reactivity to emotional faces [22,23], we also extracted betas for these two regions in the contrast Gender > Face/Place using AAL anatomical atlas masks. Extracted betas were entered into a 2 (Culture: EA, AA) × 2 (Gene: carrier, non-carrier) ANOVA to assess for main effects and interaction effects. Follow-up simple effects analyses were performed with two-tailed *t*-tests, with significance threshold set to 0.05.

## 3. Results

### 3.1. Behavioral Results

Behavioral data were collected by the same software presenting images in the scanner. Overall, there was a main effect of Culture on response time, such that the AA group responded faster than the EA group (see Table 1A for response time results). This main effect was driven by the Face/Place, Like/Dislike, and Gender trials, such that AA participants responded faster on these trials than the EA participants, but there was no group difference for the In/Out trials. There was no significant main effect of Gene, or Gene × Culture interactions for any of the trial types.

There was no significant main or interaction effects in the overall accuracy rates (see Table 1B for accuracy results). There was also no main or interaction effects for the accuracy of the Face/Place or Gender trials, nor was there a main or interaction effect in the percentage of faces that subjects reported liking in the Like/Dislike trials. There was a significant main effect of Gene (but not a Gene × Culture interaction) for the In/Out trials, such that AA carriers were more accurate than EA carriers (*t*(17) = 2.28, *p* = 0.036), whereas the AA non-carriers and EA non-carriers were not significantly different (*t*(18) = 1.47, *p* = 0.158).

### 3.2. Functional MRI Results

The whole brain results are summarized in Table 2.

#### 3.2.1. Implicit Emotional Processing (Gender > Face/Place Contrast)

Brain activation in the left amygdala, *F*(1, 33) = 8.79, *p* = 0.006), right amygdala, *F*(1, 33) = 4.78, *p* = 0.036), and left hippocampus, *F*(1, 33) = 5.00, *p* = 0.032 was significantly greater for the AA than the EA group (main effect of Culture) during implicit emotional processing (left amygdala: AA *M* = 0.95, *SD* = 0.52; EA *M* = 0.25, *SD* = 0.88; right amygdala: AA *M* = 1.03, *SD* = 1.20; EA *M* = 0.35, *SD* = 0.70; left hippocampus: AA *M* = 1.03, *SD* = 1.02 EA *M* = 0.22; *SD* = 1.09). There was also an effect of Gene in the right fusiform gyrus, *F*(1, 33) = 5.68, *p* = 0.023. Activation of this area was larger among *DRD4* 2 or 7-repeat alleles non-carriers (*M* = 1.75, *SD* = 0.73) than carriers (*M* = 1.11, *SD* = 0.61).

**Figure 1:**
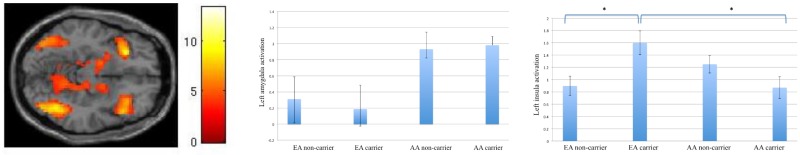
**Left**: whole brain activation for the Gender > FacePlace contrast. In this image, the insulas, fusiform gyri, and amygdalae are visible. **Middle**: graph shows betas for this contrast extracted from left amygdala. **Right**: graph shows betas for this contrast extracted from left insula. AA: Asian-born Asian; EA: European American. * *p* < 0.05 (Y axis represents beta values).

During implicit emotional processing, there was a significant Gene by Culture interaction effect in the left insula, *F*(1, 33) = 11.62, *p* = 0.002. Activation for EA carriers (*M* = 1.60, *SD* = 0.61) was larger than EA non-carriers (*M* = 0.90, *SD* = 0.49, *p* = 0.011) and AA carriers (*M* = 0.087, *SD* = 0.53, *p* = 0.014; Figure 1).

#### 3.2.2. Attention-Related Emotion Regulation (In/Out > Gender Contrast)

**Figure 2:**
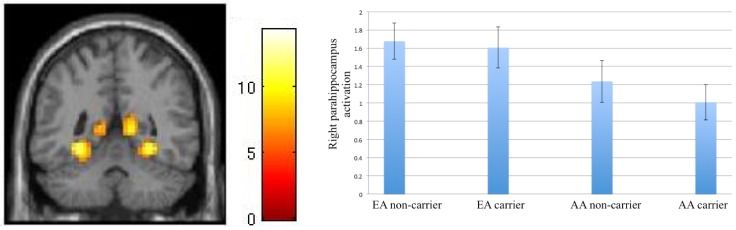
**Left**: whole brain activation for the In/Out > Gender contrast. In this image, the posterior cingulate cortex and parahippocampi are visible. **Right**: graph shows betas for this contrast extracted from right parahippocampus. AA: Asian-born Asian; EA: European American. Activation in EA participants is significantly larger than AA participants.

The only significant finding during attention-related emotional regulation was an effect of culture in the right parahippocampus, *F*(1, 33) = 5.58, *p* = 0.024. Activation in this area was larger among the EA (*M* = 1.64, *SD* = 0.65) than AA group (*M* = 1.13, *SD* = 0.65), irrespective of Gene status (Figure 2).

#### 3.2.3. Appraisal-Related Emotion Regulation (Like/Dislike > Gender Contrast)

Brain activation in this contrast did not survive the whole brain FWE correction.

## 4. Discussion

In this work we aimed to examine the effects of culture, and the interaction effects of culture and *DRD4* gene polymorphism on brain activations during the implicit emotional processing of social cues, attention-related emotion regulation, and appraisal-related emotion regulation in Asian-born Asians and European Americans. We tested for the hypothesis that carriers of the *DRD4* polymorphism would show more culture-congruent emotional processing.

### 4.1. Gene × Culture Interaction

During implicit emotional processing (Gender > Face/Place), we found a Gene by Culture interaction in the left anterior insula, such that EA carriers had a larger response than AA carriers, and EA non-carriers. This is the most intriguing finding of this study. The anterior insula is involved in the interoceptive awareness of bodily sensations, awareness of one’s emotions, as well as empathy [24]. More emotionally expressive people show a closer correlation between the strength of experienced emotions and activation in insula [11], and insula is found to have higher activation in European Americans compared to East Asians [10].

We conjecture that because experience and expression of emotions are more valued and rewarded in Western cultures, EA carriers who are more socially attuned and “norm-sensitive”, develop better awareness of their own emotional responses than EA non-carriers. On the other hand, for AA carriers to be culturally-congruent, they should be more suppressive of their own emotional reactivity, and hence show smaller response in the insula. Our behavioral finding that AA carriers were more accurate during In/Out trials is also suggestive of greater success in attentional regulation of their emotional responses, and higher ease (especially in the context of their generally faster response time) with shifting attention away from emotional faces to the background scene. However, this finding needs to be replicated in future studies, as it is based on a small number of participants in each cell.

### 4.2. Effect of Culture

Asian-born Asian, compared to European American, participants showed larger activation in the left amygdala, right amygdala, and left hippocampus during implicit processing of social emotional cues (Gender > Face/Place). This finding is quite instructive and potentially very interesting.

The amygdala is involved in the salience detection of incoming stimuli and emotional reactivity [25,26]. The hippocampus is also involved in contextual processing and understanding the emotional stimulus in its physical and temporal context [27]. Our finding suggests that fearful faces are more salient to AA participants. In an interdependent culture, emotion has a more relational role and the person should be sensitive to and take perspective of the other, to be able to maintain a connection to the group [3]. Furthermore, in Asian cultures where emotions are more commonly suppressed and less expressed, their expression may be more salient and cause a larger amygdala reactivity. In other words, if a signaling threat happens less commonly, its presentation may cue a larger threat, and cause a greater amygdala response. An alternative explanation could be based on some evidence that humans may show larger amygdala response to viewing outgroup faces [28]. In a study of white and black participants viewing ingroup and outgroup faces, outgroup faces elicited greater amygdala reactivity only during the second half of the study [29]. However, other studies have found larger brain response to pictures of ingroup members especially when observing ingroup suffering or negative emotions. In a prior study of European American and Japanese participants, Chiao and colleagues found larger left and right amygdala and hippocampal response to ingroup fearful faces compared to outgroup fearful faces, but there was no difference in response to angry, happy, or neutral faces [30]. Other studies have reported increased orbitofrontal cortex (OFC) activation and connectivity between the left OFC and amygdala [31], as well as larger activation in left temporoparietal junction [32] in response to pictures of suffering inflicted on ingroup than outgroup members. Together, these findings suggest that it is less likely that ingroup/outgroup differences could explain culture specific effects in our study as in that case, one would expect larger amygdala activation in EA participants (as the pictures are ingroup for these participants). Furthermore, we used neutral Caucasian faces as a comparison in this contrast (Gender > Face/Place), and this may have removed the main effect of the ethnicity of the faces and have only left the threat (fear) of specific effects. An alternative explanation however may exist: as AA participants are more likely to be affected by the background “contextual” information, during this task they may have are stronger implicit processing of the background information (here the emotion of the faces).

We also found an effect of culture in the In/Out > Gender contrast such that the EA participants had a larger response than the AA participants in the right parahippocampus. To explore the cause of this effect, we examined group difference in the Gender>baseline contrast, and found larger activation in the same area among AA participants. It is thus likely that the effect observed in the In/Out>Gender contrast is contributed by the larger response in this area in AA participants during implicit emotional processing, consistent with the pattern seen in nearby regions of amygdala and hippocampus, discussed in previous paragraph.

### 4.3. Effect of Gene

During implicit emotional processing (Gender > Face/Place), activation in the right fusiform gyrus was larger among non-carriers of *DRD4* polymorphism. Fusiform gyrus is involved in facial recognition processing [33,34]. In the light of our hypothesis, it is possible that carriers of *DRD4* polymorphism, who are more socially attuned, require less processing resources in this area for the processing of social cues. That may explain why both AA and EA participants who are carriers show reduced activation of this area when asked to engage in processing of face stimuli. In other words, *DRD4* carriers do this task less effortfully.

This is the first work that examines culture and gene interactions and the role of nature and nurture in the formation of emotional responsivity to social cues and its neurocircuitry. To summarize, our findings suggest that differences in culturally rewarded social behavior may shape perception of social signals as more salient to Asians who are from more interdependent cultures that reinforce attunement to others’ emotions, and limited expression or suppression of emotions. This is evident in the larger activation in the amygdala and hippocampus in their response to emotional faces. Also, carriers of the *DRD4* variant gene seem to acquire their cultural specific norms more strongly than the non-carriers. European-American *DRD4* polymorphism carriers, whose cultural rewards experience and expression of individual emotions seem to be more aware of their emotional response to the social cues (higher activation in insula). On the other hand, Asian-born *DRD4* carriers who acquire their cultural norm of suppressing one’s emotional response have a lower activation in the same insular area.

This study has several limitations. First, while the overall number of participants (39) is acceptable for neuroimaging study, the number of participants in each of the four groups (9 to 10) is low, and hence the interaction finding should be considered cautiously and needs to be replicated in future. We believe this work is however important as there is little work in this area that is present in the literature. Studies with a larger number of participants are needed to replicate our findings. There were also a higher number of female participants among carriers in both groups, relatively due to a higher number of female participants in the parent study. The proportion of female/male participants in the groups did not differ; however, we also controlled for gender in our analysis. Some of the effects might be contributed by gender differences, and we did not have a way to control for it or to study it further due to the small numbers of participants in sub-groups. Second, all the faces presented in this study were Caucasian, and thus we cannot completely rule out the possibility that culture differences in reaction to ingroup/outgroup facial features may have contributed to our culture specific findings. Third, because of the nature of the Gender > Face/Place contrast, we could not assess the intensity of the emotional response and its correlation with brain activation. Because this contrast is meant to examine implicit emotional reactivity, we could not ask participants to report the intensity of their emotional reaction to the faces. As a result, we could not assess the subjective level of emotional reactivity and correlate it with the functional neuroimaging findings. Also, cognitive processing could be contributing to the “Gender” task when the participants are instructed to identify the gender of the faces. We try to control for this cognitive processing by using the control condition, which uses the identification of human “Faces” as a baseline, and hence we believe the majority of the activation here is contributed by implicit emotional processing. Our previous studies on this task have consistently shown the activation of implicit emotion processing areas. However, cognitive processing may still contribute to emotional processing in this task. Finally, in this study, participants were only exposed to simple facial expressions, which limits the generalization of the findings to real life situations where people are presented with a multitude of other social cues (such as language and tone of voice), and contextual information. More work is needed to address these limitations. The present findings serve as further impetus to examine the interplay of gene and culture to gain a better understanding of systematic differences in not only emotional processing, but processing and behavior across a broad range of domains.

## Figures and Tables

**Table 1:** (**A**) Response time results (reported in milliseconds). (**B**) Accuracy results.

(**A**)							
	**EA Carriers M (SD)**	**EA Non-Carrier M (SD)**	**AA Carriers M (SD)**	**AA Non-Carrier M (SD)**	**Ethnicity Main Effect F (*p*)**	**DRD4 Main Effect F (*p*)**	**G × C Interaction F (*p*)**
Overall Average	1100.97 (165.13)	1174.47 (103.93)	995.76 (130.20)	1073.55 (108.49)	7.48 (0.010)	3.54 (0.068)	0.01 (0.981)
FacePlace	885.28 (157.18)	919.46 (113.77)	772.60 (90.98)	784.30 (92.22)	11.46 (0.002)	0.26 (0.611)	0.29 (0.594)
In/Out	1135.93 (165.89)	1226.04 (176.98)	1062.22 (121.27)	1169.28 (188.37)	1.65 (0.208)	4.14 (0.050)	0.01 (0.937)
Like/Dislike	1181.83 (207.51)	1302.06 (120.73)	1059.04 (219.70)	1172.43 (193.32)	4.16 (0.049)	3.33 (0.077)	0.01 (0.949)
Gender	1200.82 (184.12)	1250.30 (115.95)	1089.18 (166.91)	1168.18 (86.08)	5.45 (0.025)	2.96 (0.094)	0.11 (0.738)
(**B**)							
	**EA Carriers M (SD)**	**EA Non-Carrier M (SD)**	**AA Carriers M (SD)**	**AA Non-Carrier M (SD)**	**Ethnicity Main Effect F (*p*)**	**Gene Main Effect F (*p*)**	**G × C Interaction F (*p*)**
Overall Accuracy Average	0.73 (0.10)	0.73 (0.06)	0.77 (0.05)	0.71 (0.05)	0.36 (0.554)	1.76 (0.193)	1.75 (0.195)
FacePlace Accuracy	0.99(0.03)	0.97 (0.07)	0.99 (0.01)	0.98 (0.03)	0.46 (0.502)	1.27 (0.268)	0.21 (0.650)
In/Out Accuracy	0.82 (0.09)	0.83 (0.09)	0.90 (0.06)	0.77 (0.10)	0.07 (0.795)	4.71 (0.037)	6.06 (0.019)
Gender Accuracy	0.69 (0.13)	0.67 (0.12)	0.73 (0.06)	0.71 (0.09)	2.61 (0.116)	0.04 (0.847)	0.03 (0.873)
% Like	0.56 (0.17)	0.58 (0.12)	0.64 (0.17)	0.67 (0.17)	2.86 (0.099)	0.03 (0.873)	0.31 (0.583)

**Table 2:** Areas of significant brain activation among all participants at whole brain FWE corrected *p*-value threshold level 0.05 and minimum cluster size of 50 voxels (areas of activation in the occipital lobe are excluded). Coordinates are in MNI system.

Area of Activation	Z Score	Cluster-Level FWE Corrected *p*-Value	Cluster Size	x	y	z
**Gender > FacePlace**						
Anterior cingulate	6.37	<0.001	346	6	26	34
Left insula	7.59	<0.001	151	−30	26	−5
Right insula	6.57	<0.001	230	33	29	−5
Left fusiform	6.20	<0.001	158	−36	−67	−23
Right fusiform	7.93	<0.001	566	39	−64	−17
Left caudate	6.05	<0.001	67	−12	−1	7
**In/Out > Gender**						
Left posterior cingulate	7.52	<0.001	115	−15	−58	16
Right posterior cingulate	7.79	<0.001	154	18	−55	19
Left parahippocampus	8.04	<0.001	220	−30	−43	−11
Right parahippocampus	7.99	<0.001	206	33	−40	−11
**Like/Dislike > Gender**						
None						

## Supplementary Materials

The following are available online at http://www.mdpi.com/2076-328X/8/7/62/s1, Figure S1: SEAT Task: There were 3 runs and a total of 120 trials (30 Gender, 30 In/Out, and 30 Like/Dislike. The remaining 30 trials were pictures of a neutral face or a place, and participants were asked to determine if it was a picture of a face or a place (Face/Place). Trials were presented in random order and consisted of presentations of a crosshair for 2000–5000 ms, a task cue for 750 ms, and a composite image for 1500 ms.
