# Supplementary figures and images for: Gene by Culture Effects on Emotional Processing of Social Cues among East Asians and European Americans
Source: Behav Sci (Basel). 2018 Jul 11;8(7):62. doi: 10.3390/bs8070062 (PMC6070862; doi:10.3390/bs8070062)

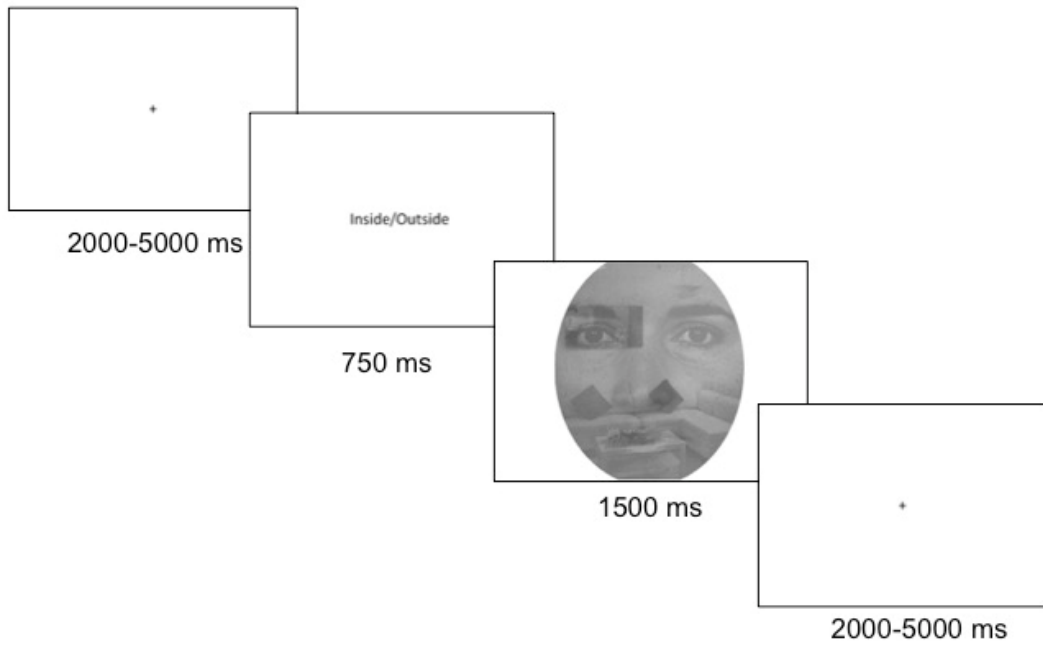

Supplement: Supplementary file 1 [file behavsci-08-00062-s001.pdf]
